# Effect of infestation with *Psoroptes cuniculi* on reproduction and behavior of obese rabbit does (Oryctolagus cuniculi)

**DOI:** 10.1371/journal.pone.0307803

**Published:** 2024-08-28

**Authors:** Guadalupe Arjona-Jiménez, Iván Flores-Pérez, José Benigno Valdez-Torres, Yazmin Briseida Jiménez-Nevárez, Armida Báez-Saldaña, Mariana Pedernera, Claudia Hallal-Calleros

**Affiliations:** 1 Facultad de Ciencias Agropecuarias, Universidad Autónoma del Estado de Morelos, Cuernavaca, Mexico; 2 CIAD A.C. Coordinación Culiacán, Culiacán Rosales, Mexico; 3 Instituto de Investigaciones Biomédicas, Universidad Nacional Autónoma de México, Mexico City, Mexico; Beni Suef University Faculty of Veterinary Medicine, EGYPT

## Abstract

Parasites and obesity are health problems worldwide. Rabbits are production animals yielding one of the healthiest meats, also taking advantage of skin, hair, limbs and excreta. It is among the most frequent pets in some countries and widely used as animal model in research. *Psoroptes cuniculi* is a mite of high transmission rate, affecting welfare and production and obesity causes multiple metabolic, endocrine and immunological disorders, being an emerging problem in domestic animals. Obesity and acarosis are prolonged stressors, modifying the activity of the hypothalamic-pituitary-adrenal axis that can induce metabolic and behavioral disorders. Alterations caused by comorbidities could be similar to or different from those induced by each morbidity separately. We analyzed the influence of obesity on the infection degree with *P*. *cuniculi* and on behavior and production. Rabbit does induced to obesity were infected and mated; behaviors in the open field test, obesity estimation indices and productive parameters at delivery and weaning were analyzed. The acarosis induced a decrease in feed intake and a decrease in body weight, a decrease in locomotor, exploratory and chinning behaviors in normal weight and obese does. The infection induced 23% mortality at birth, obesity 45% and comorbidity 74%, while in normal weight rabbits a 6.5% was observed. Weight gain from birth to weaning was lower in the comorbid group, reaching a litter weight of 4.5±0.13 kg in healthy normal weight does and 2.6±0.67 kg in comorbid does. The disturbances induced by the comorbidity were magnified in both behavioral and productive parameters.

## Introduction

Rabbits represents one of the most interesting production animals as they can be considered an ideal meat producing animal; have a short life cycle, are very prolific, have a short gestation period, and a high feed conversion capacity; Its meat is a healthy food as it is rich in protein while low in fat, cholesterol, and sodium [1]. Also, European rabbits have been used in research since the middle of the 19th century, they have been used as a model of human pregnancy, atherosclerosis, osteoporosis, surgical implantation of biomedical devices, pharmacologic studies for teratogenicity testing of novel pharmaceutic compounds, ocular and immunology research. Furthermore, rabbits are between the most prevalent pets, reaching the third place in some countries, after dogs and cats [2]. Parasites and obesity on the health of humans and animals are well recognized problems and rabbits are not the exception, as they are susceptible to many of the same problems caused by parasitosis and obesity in other species. There are, however, some conditions that are more rabbit specific. Among the parasitosis that affect rabbits, the acarosis caused by *Psoroptes cuniculi* is the most frequent dermatological disease, it has a high transmission rate affecting welfare and production cycles [3–5]. *Psoroptes cuniculi* is a common worldwide parasite that causes considerable weight loss, lower feed conversion rates, vestibular dysfunction, and meningitis, frequently complicated by secondary bacterial infections leading to dead seriously endangering the healthy development of rabbit production industry [6] (Shang et al. 2014). It parasitizes the external auditory canal, and being the fastest animal in relation to its size, makes it difficult to accurately assess the parasite load either by qualitative or quantitative methods [7–9].

Obesity causes multiple vascular, metabolic, endocrine and immunological disorders, being an emerging problem in domesticated animals including rabbits [10], in which, the adverse effects of obesity have not yet been characterized in detail. Therefore, as with other companion animal species, regular weight and body composition assessment should be considered as part of general health status monitoring in rabbits. Measure of adipose tissue mass body condition is a challenge in any animal species, although there are several methods for determining body composition in animals. Dual-energy x-ray absorptiometry is a relatively reliable research technique but can be expensive and not practical for routine use in primary care practice. Body weight is simple to measure, can be precise and accurate, but it correlates poorly with body composition. The Quetelet index, known as body mass index, associates the weight with the height of an individual and is the most widely accepted criterion in humans, although it is still cause of controversial discussions, considering that the waist-height index is a better indicator of pathologies related to obesity. In animals, inaccuracies are even greater, with body condition being a popular measure estimated using visual and palpable characteristics, being unreliable when used by non-professionals or individuals without enough training [11]. Body condition scoring systems have been used for rabbits, although to date, these have not been properly validated in this species. A zoometric index has been theorized by Sweet et al. [10] found to be accurate for medium sized rabbits that it is worth further validation.

The obesity-parasite interaction may be a prolonged stressor, suggesting that the activity of the hypothalamic-pituitary-adrenal axis may chronically modify activity patterns, causing metabolic and behavioral disorders [12, 13], which could be similar to or different from those that could be induced by each of the morbidities separately. In mice experimentally infected with the nematode *Nippostrongylus besiliensis*, it was reported that the parasitism had opposite effects to what was expected, since with the infection, obesity decreased, implying a lower risk of suffering from type 2 diabetes [14]. In other studies, it has been described that obesity-parasitosis comorbidity alters behavior in male rabbits infected with *Taenia pisiformis*, where locomotor, exploratory, chinning and sexual behaviors are reduced, also affecting productive and reproductive parameters [15]. The consequences of the alterations caused by the parasitosis-obesity comorbidity have not been clearly elucidated in rabbits infested with mites. The objective of this work was to determine if obesity influences the degree of infection with *P*. *cuniculi*, and if the presence of the mite and obesity modify characteristic behaviors of this species and important productive parameters.

## Materials and methods

### Ethical considerations

This work was developed in accordance with the official Mexican standards [16] and international guidelines ARRIVE 2.0 [17], approved by the research ethics committee of the Universidad Juárez Autónoma of Tabasco (approval sheet 1005).

### Experimental groups

Twenty-eight nulliparous New Zealand does of 2.7±0.2 kg were divided into two groups of 14. Does were housed individually in 60 X 90 X 40 cages and provided with water in automatic drinkers on demand throughout the experiment. The normal weight group (NW) was fed with a maintenance diet, consisting of pellets for adult rabbits (Ganador®, Malta Cleyton, Mexico, 16% protein, 3% fat and 17% fiber), providing them with 180 g per day according to with the recommendations of Lebas and Laplace [18]; the obese group (OB) was fed on demand with maintenance diet added with 5% soybean oil and 5% lard throughout the experiment [15]. Once the OB group reached 17% more weight than the NW (after 56 days of diet), both groups were subdivided into 2 groups of 7 does each. Seven does from the normal weight group (NW) and 7 from the obese group (OB) remained uninfested, and seven does from the normal weight group (iNW) and 7 from the obese group (iOB) were infested with *P*. *cuniculi*. At 7 days post-infestation, all the does were mated using sexually experienced bucks, receiving two mattings each. From the day of mating, the four groups of rabbits were fed on demand.

### Voluntary feed intake

Was measured every 7 days, offering 400 g of pellets in each feeder and subtracting the weight of the residual feed 24 h later.

### Obesity estimation indices

The body weight was recorded weekly using a digital scale. The zoometric index (ZI) and body mass index (BMI) were evaluated every 7 days as reported by Sweet et al. (2016); briefly, distal forelimb length (DFL) was measured with an anthropometric tape from the lateral surface of the olecranon to the dorsal surface of the distal edge of the middle finger (digit two), and vertebral length (VL) from the base of the occiput to the sacrocaudal junction, following the curvature of the spine. The following formulas were applied: BMI = Body weight (kg)/ DFL (cm); ZI = Body weight (kg)/VL (cm).

### Open field test

Individual behavior was evaluated for 10 minutes by placing a female rabbit in the lower middle quadrant of a 1.20 x 1.20 m arena divided into 9 quadrants, in which three bricks were placed in the upper left quadrant [4]. Locomotor activity was evaluated considering the number of times the rabbit passed from one quadrant to another; exploratory activity was recorded as the number of times the rabbit got up on the hindquarters; chinning behavior was assessed by counting the number of times the rabbit rubbed her chin on the bricks. The observations were performed every third day, being suspended only for two weeks before and two weeks after birth, caring for the welfare of the does.

### Infestation with *P*. *cuniculi* mites

The does of the corresponding groups were infested by placing 150 mites in the intra auricular pavilion of each ear, fixing them with a piece of cotton and adhesive tape during 6 days [4].

### Productive parameters at delivery and weaning

On the day of birth, the size and weight of the litter were recorded using an anthropometric tape and a digital scale, and the number of young rabbits born (alive and dead) was registered. At weaning (28 days postpartum) the number of rabbits and the weight of the litters were recorded.

### Assessment of *P*. *cuniculi* infestation

The infestation was estimated qualitatively and quantitatively. The determination of the degree of qualitative infestation was made by observing lesions or scabs on the auricle of each rabbit, observing the ear and the auditory canal with an otoscope (Checktec®), where 0 = absence of crusts and mites, 0.5 irritation of the ear canal without observation of crusts or mites, 1 = few mites in the ear canal, 2 = little crusts with mites 3 = crusts with mites in ¼ of the auricle; 4 = scabs with mites on half of the pinna; 5 = with mites in ¾ of the ear; 6 = pinna crusted over with mites [7, 9]. Scores were assigned by grouping 0 and 0.5 as absent, 1 and 2 as low, 3 and 4 as medium, and 5–6 as high. For the quantitative evaluation, at day 63 post infestation, the does were humanely sacrificed in accordance with animal welfare standards, by applying sodium pentobarbital in a lethal dose of (100 mg/kg), prior anesthesia with xylazine/ketamine (5/35 mg/kg) [19]. The extension of the lesion was measured by using a 10x7 cm transparent plastic film, marked with a 1 cm^2^ grid, which was placed on the extended ear to observe the area of infection [9].

### Statistical analyses

The data obtained from locomotor activity, exploratory activity, chinning, body weight, BMI, ZI, and feed consumption were analyzed by ANOVA of repeated measures, followed by a multiple comparisons Tukey post-hoc test; Kruskal-Wallis test was used when data did not meet the assumptions of normality, followed by Dunn´s test. For the degree of infection and the area of the lesion the paired student’s T test was used; data is expressed as Mean±SE, with statistical significance at P<0.05, analyses were performed using the GraphPad Prism 8.0 statistical program.

## Results

### Voluntary intake and weight gain of does fed a balanced or obesogenic diet

**Fig 1:**
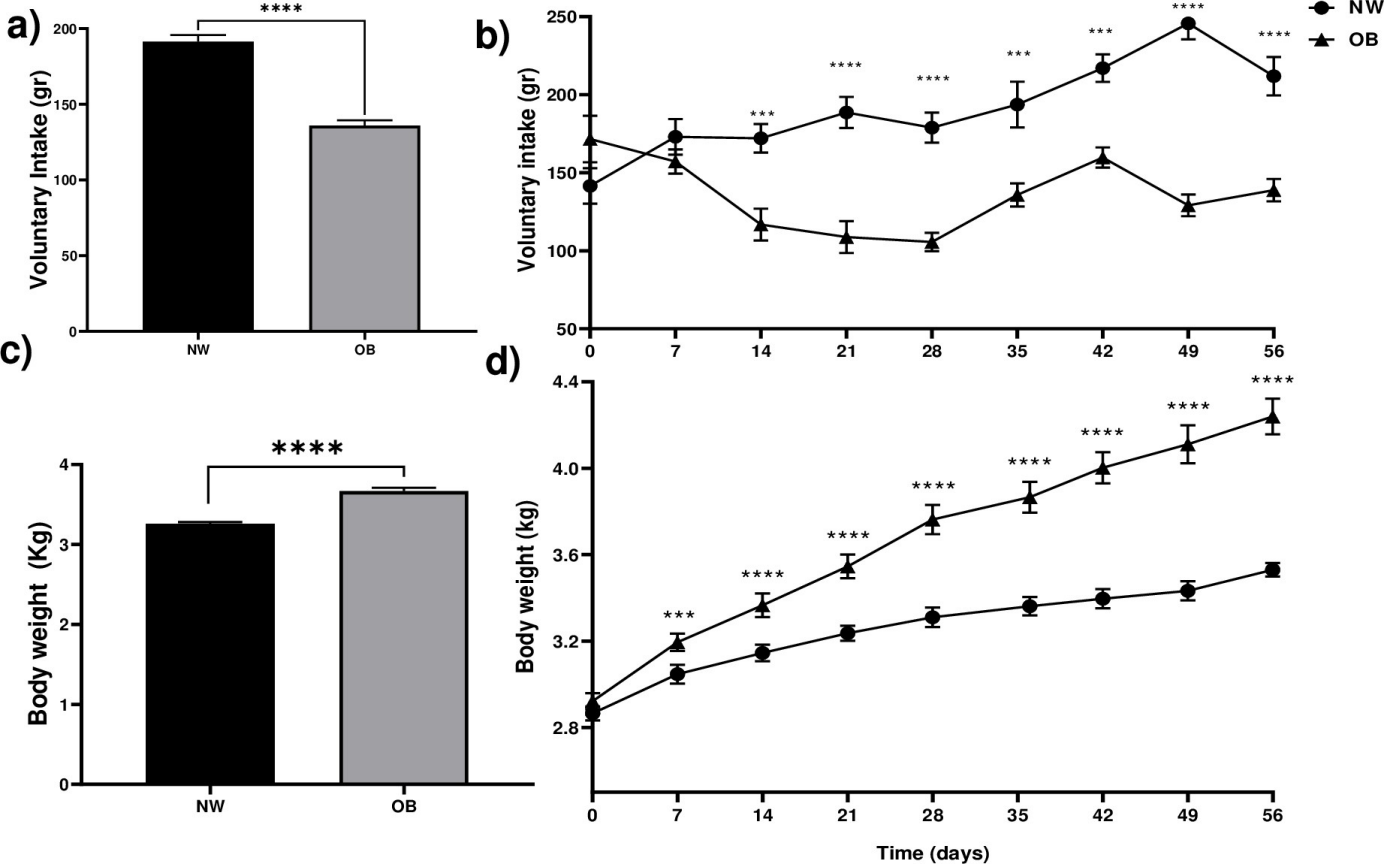
Obesity induction in rabbits. a) Total voluntary intake for 56 days. b) Voluntary intake over time. c) Total body weight of the rabbits for 56 days. d) Body weight of the rabbits over time. NW = normal weight; OB = obese. Mean±EE, ***P≤0.001, **** P≤0.0001 a) and c) repeated measures ANOVA, Tukey post-hoc test. Asterisks in b) and d) show differences between groups compared on the same day, paired students T-test.

During the induction to obesity, the does in the group fed with the obesogenic diet (OB) consumed a total of 29% less feed during the obesity induction time, compared to the normal weight group (NW) (Fig 1A). From day 21, a 43% decrease was observed in the OB group with respect to the NW (109 gr vs 189 gr), maintaining a lower consumption in the OB group throughout the evaluation time. At day 49, the decrease in feed consumption in the OB group reached 48% (129 vs 246, Fig 1B). Despite the lower consumption of OB rabbits, the group reached a higher weight after 7 days of diet. The difference in weight increased over time, reaching a 17% increase on day 56 after the start of the diet, compared to the NW group (4.2 vs 3.5 kg, Fig 1C and 1D).

**Fig 2:**
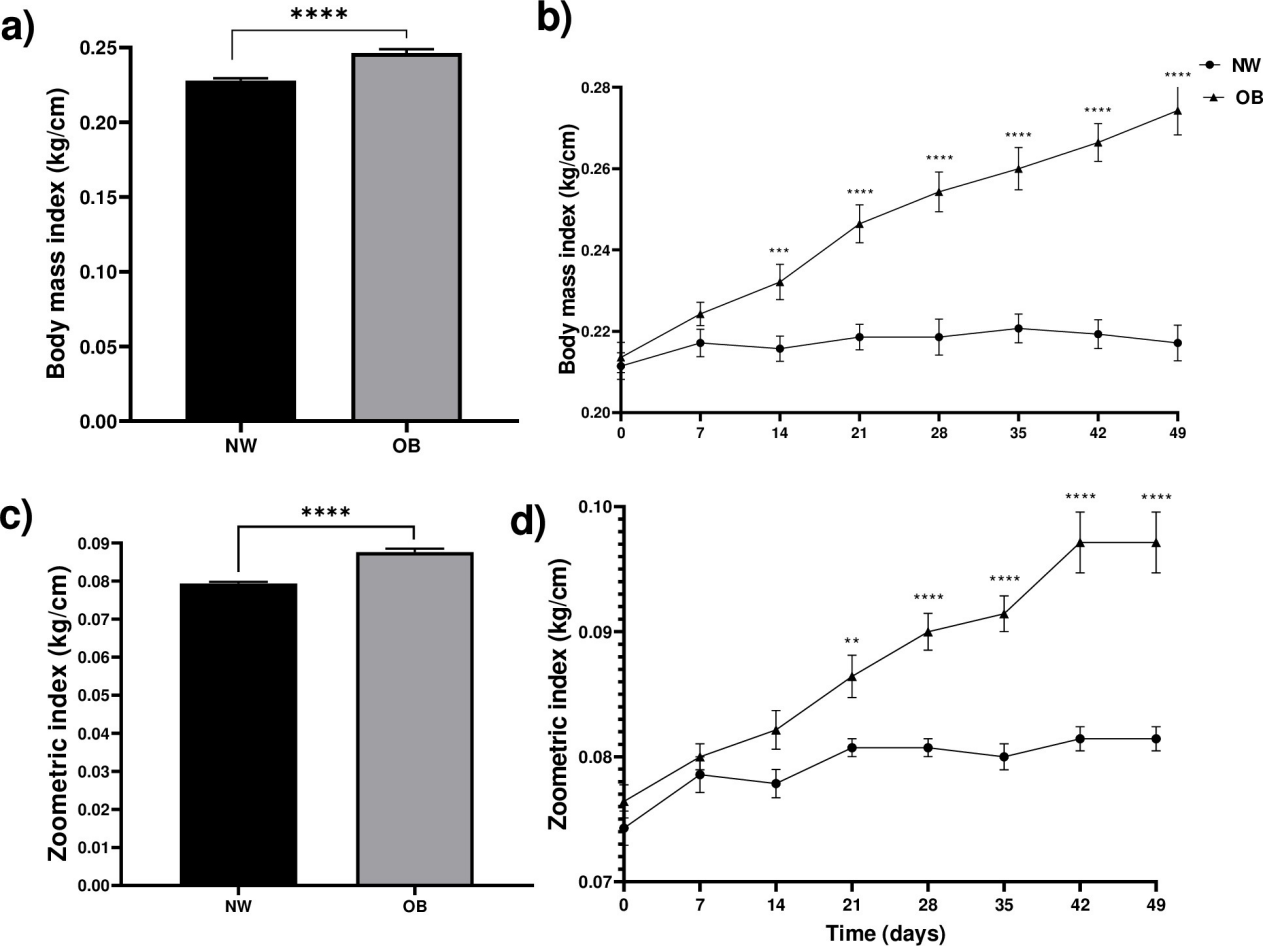
Obesity measurements in rabbits. a) Total body mass index for 49 days. b) Body mass index over time. c) Total zoometric index for 49 days. d) Zoometric index over time. NW = normal weight, OB = obese. Mean ±EE, ***P<0.001, ****P<0.0001. a) and c) repeated measures ANOVA, Tukey’s post-hoc test. Asterisks in b) and d) show differences between groups compared on the same day, paired students T-test.

The body mass index (BMI) was higher in OB than in NW animals. The changes between the two groups were observed after 14 days, finding a higher value in the OB group compared to the NW group; the increase in the OB group was higher over time, observing 22% more (0.05 kg/cm) compared to the NW group on day 49 post-diet (Fig 2A and 2B). The zoometric index (ZI) behaved similarly to the BMI, where the OB group had a higher value, with a constant increase over time, while the NW group remained constant over time. The difference between the groups was observed after 21 days of starting the high-fat diet, where there was an increase in obese rabbits compared to NW. After 49 days of diet, the increase observed in the OB group was 25% (0.02 kg/cm, Fig 2C and 2D).

### Behavior of does fed a balanced or obesogenic diet

**Fig 3:**
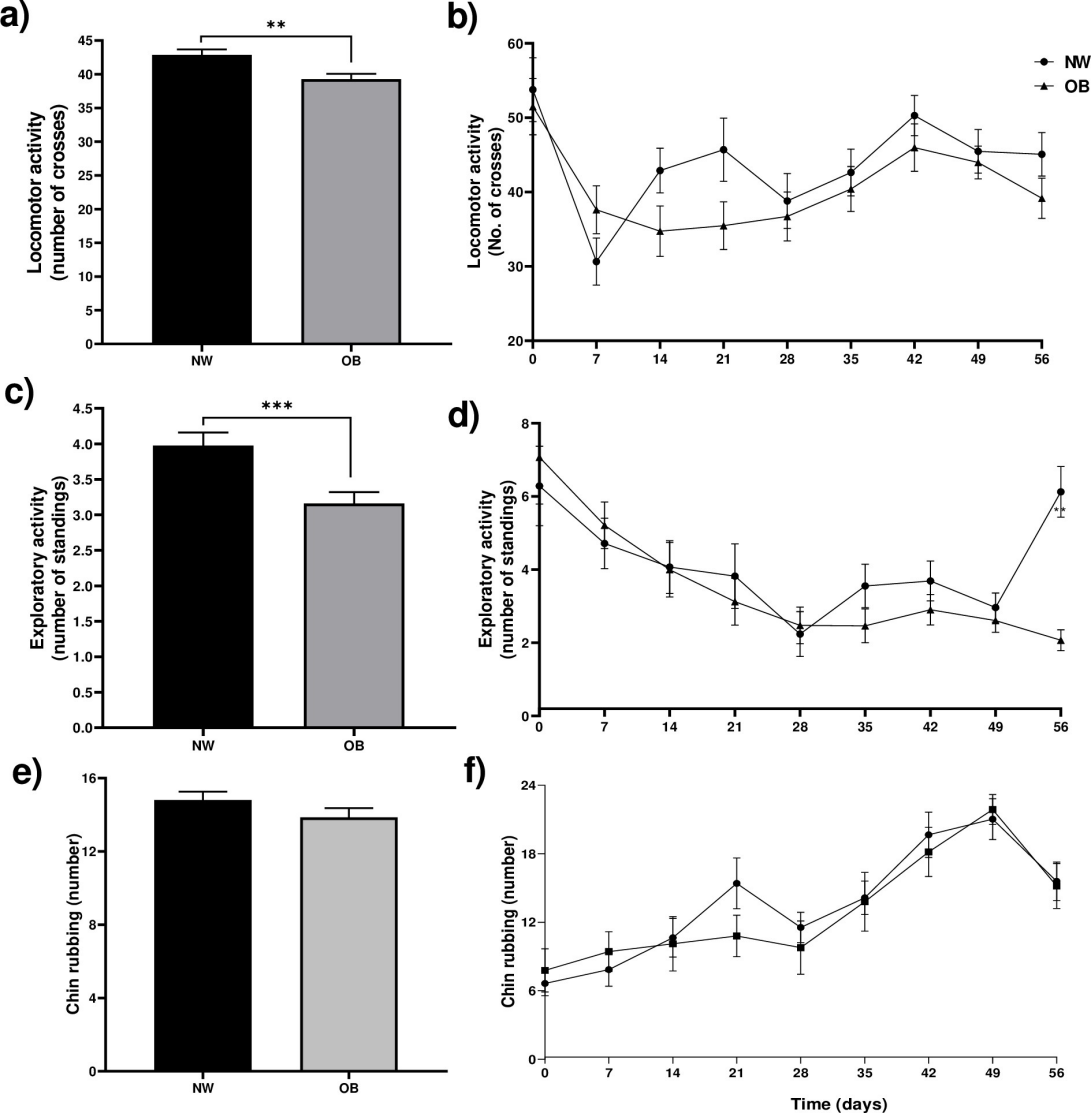
Behaviors in rabbit does in the open field test during obesity induction. a) Total locomotor activity for 56 days. b) Locomotor activity over time. c) Total exploratory activity for 56 days. d) Exploratory activity over time. e) Total chin rubbing mark for 56 days. f) Chin rubbing mark over time. NW = normal weight, OB = obese. Mean±EE of three measurements per week. **** P≤0.0001. a) and c) repeated measures ANOVA, Tukey’s post-hoc test; b) and d) paired students T-test.

In the open field test during obesity induction, a 9% decrease in locomotor activity was observed in the OB group in the accumulated values during the 56 days (Fig 3A); also, a 25% decrease in exploratory activity (Fig 3C) and no differences were observed in chinning behavior (Fig 3E). The locomotor activity, exploratory activity and chin rubbing behavior were very similar in both groups during the 56 days of consumption of the obesogenic diet (Fig 3B, 3D and 3F). At 57 days of diet (time zero), 7 of the 14 normal-weight does (iNW) and 7 of the 14 obese does (iOB) were experimentally infected with *P*. *cuniculi*, and 7 days after infestation, the 28 experimental does received 2 mounts with ejaculations of bucks.

### Voluntary intake and weight gain of does after infection

**Fig 4:**
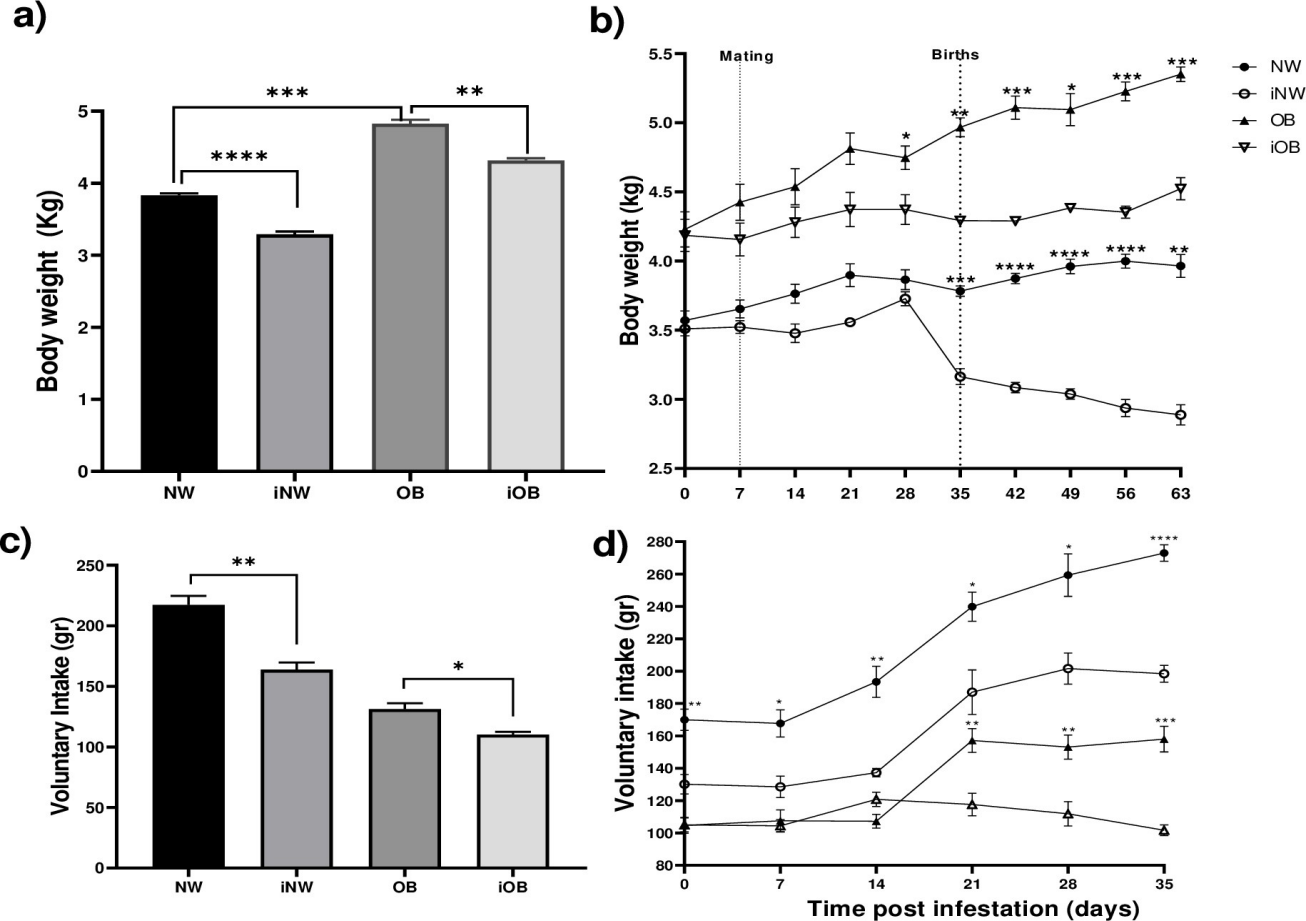
Body weight and voluntary intake in healthy or infected normal weight or obese rabbits. a) Total body weight for 63 days postinfestation with *P*. *cuniculi*, b) Body weight over time, c) Total voluntary intake for 35 days. d) Voluntary intake over time. Mean±EE, *P≤0.05, **P≤0.01, ***P≤0.001. ****P≤0.01. Repeated measures ANOVA, Tukey’s post-hoc test. Asterisks in b) and d) show differences between NW and iNW or" to "Asterisks in b) and d) show differences between NW and iNW." and proceed.

In the global analysis of body weight, the OB animals had 14% more weight than the NWs. In the iNW animals, a 13% decrease was observed with respect to the NW, and in the iOB it decreased by 10% with respect to the OB. Over time, a constant weight gain was maintained in the two groups of obese animals, but after 28 days post-infestation, the OB animals gained 400 g more (9% more) than the iOB, and at 63 days the group OB weighed 900 grams more (20% more) than iOB. The NW group had a gradual weight gain during the 63 days post infestation, while in the iNW group there was a decrease in body weight from day 35 post-infestation, which caused a weight difference of 19%, with 600 g more in the NW group. At 63 days, the difference between the two groups was 38%, with a difference of 1.1 kg between the two groups (Fig 4B). In comorbid animals, the effect on weight loss was 46%, observing a weight of 5.4 kg in the NW while in the iOB a weight of 2.9 kg was observed (Fig 4B). The voluntary intake after the infestation was measured only during 35 days to avoid ambiguities due to the possible consumption of the young rabbits (Fig 4D); we observed that both in NW and OB groups, the infection induced a decrease in consumption. The effect was more pronounced in obese rabbits, observing a 55% decrease in the iOB group (158 gr *vs* 102 gr). However, comorbidity induced a very marked decrease of 168% in consumption (273 g in NW vs 102 g in iOB).

### OB and iOB groups compared on the same day

**Fig 5:**
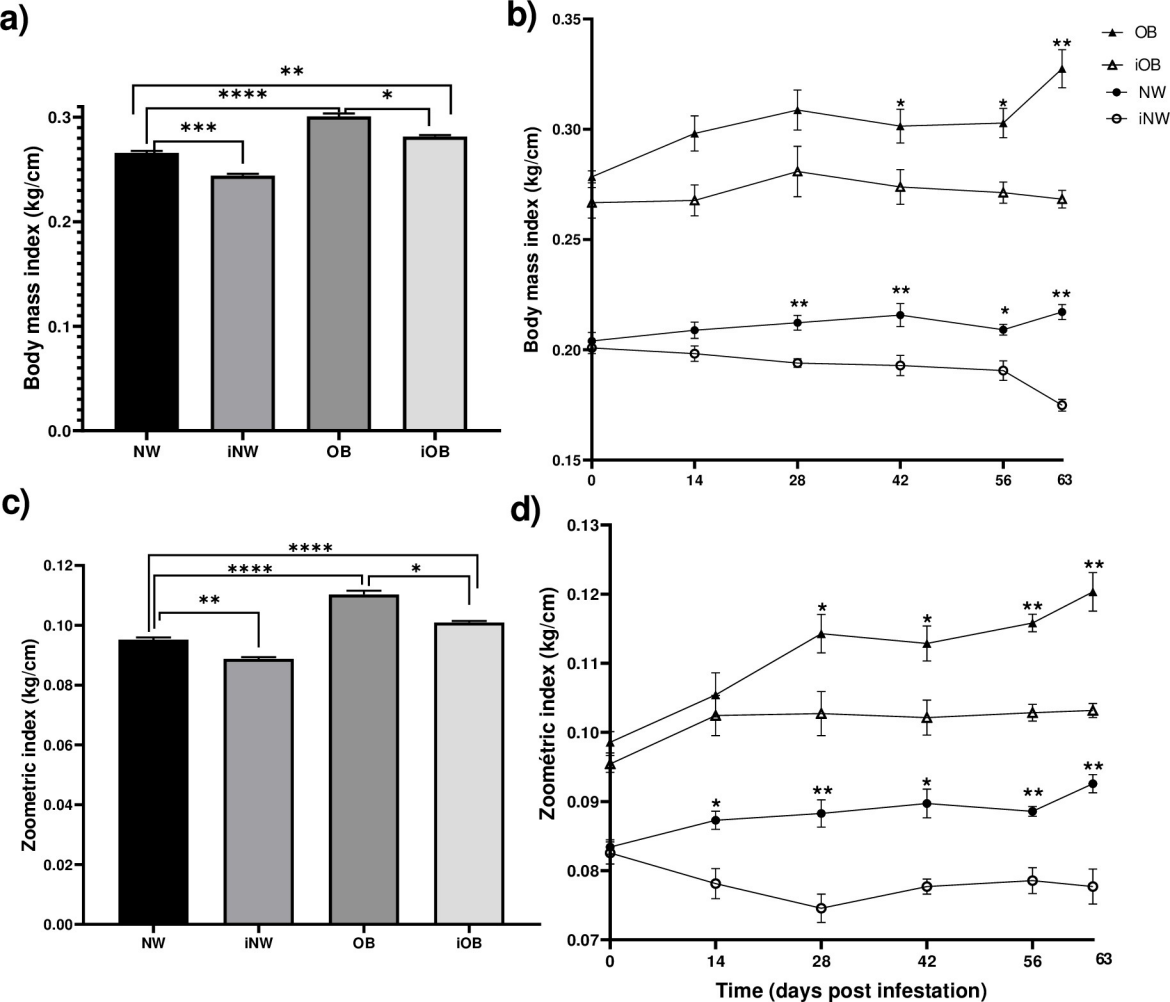
Measurements of obesity in rabbits infected with *P*. *cuniculi*. a) Total body mass index for 63 days. b) Body mass index over time. c) Total zoometric index for 63 days. d) Zoometric index over time. NW = normal weight, OB = obese, iNW = infested normal weight, iOB = infested obese. Mean±EE, *P≤0.05, **P≤0.01. Repeated measures ANOVA, Tukey’s post-hoc test. Asterisks in b) and d) show differences between groups compared on the same day.

The BMI and the ZI were higher in OB animals compared to NW, and decreased in infected animals, both NW and OB (Fig 5A and 5C). In the BMI (Fig 5B) differences were observed between OB and iOB rabbits from day 42 post-infestation (0.3 vs 0.37), and between NW and iNW from day 28 (0.21 vs 0.19). The zoometric index in the infested rabbits began to show differences at 28 days post-infestation between the OB and iOB groups and up to 63 days where a 0.2 kg/cm difference was observed. Differences were observed between the NW and iNW groups from day 14 post-infestation, with a ZI of 0.09 in NW and 0.07 in iNW at 63 days (Fig 5D).

### Behavior of does after infection

**Fig 6:**
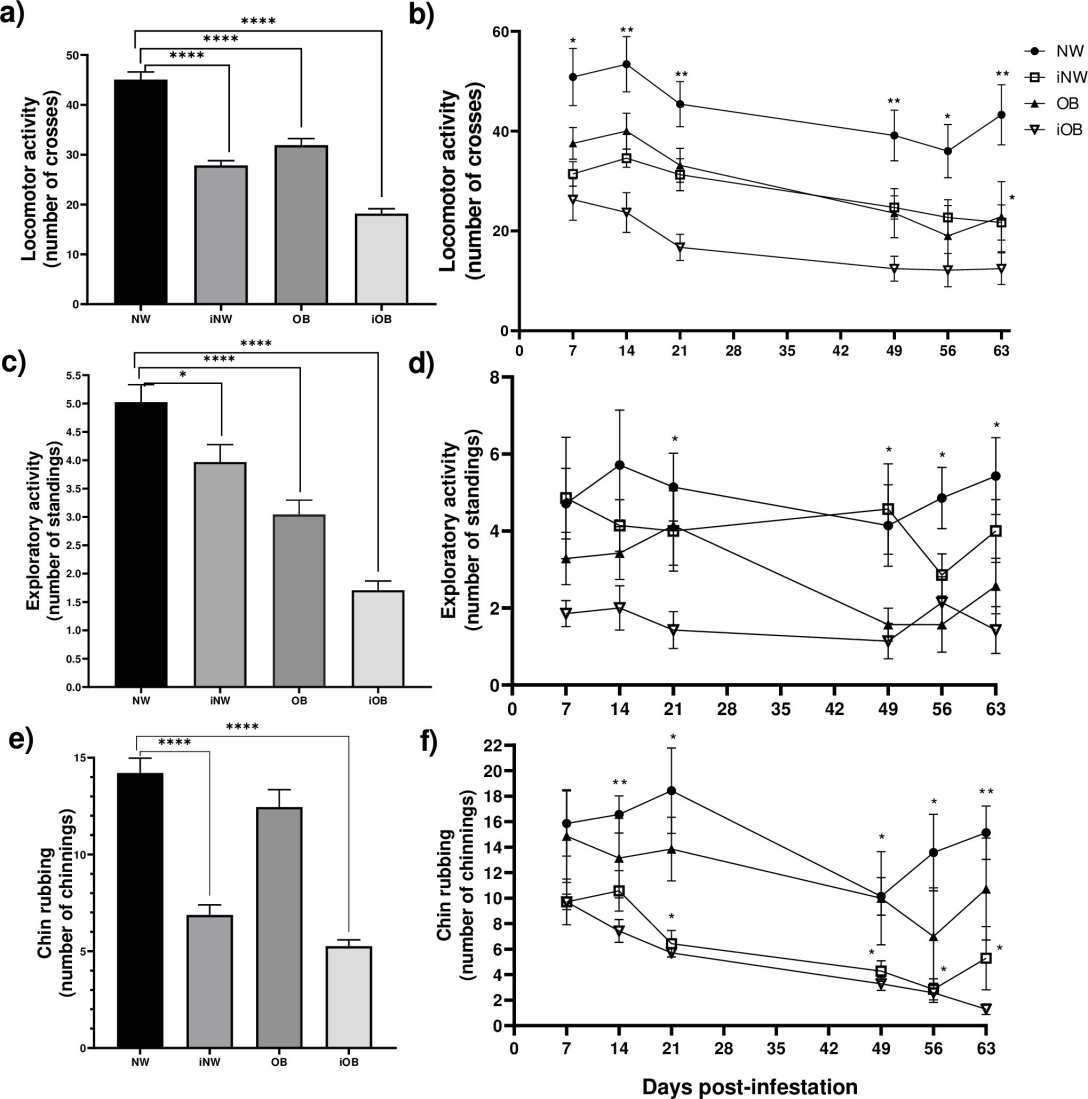
Behaviors in rabbit does in the open field test after infection with *P*. *cuniculi*. a) Total locomotor activity for 63 days. b) Locomotor activity over time. c) Total exploratory activity for 56 days. d) Exploratory activity over time. e) Total chin rubbing for 63 days. f) Chin rubbing over time. NW = normal weight, OB = obese, iNW = infested normal weight, iOB = infested obese. Media±EE, repeated measures ANOVA, Tukey post-hoc test, *P≤0.05; **P≤0.01. b) and d) show differences between NW and iNW or OB and iOB groups compared on the same day.

In the behavior analyzed during the open field test after infection, locomotor activity and exploratory activity decreased in iNW animals and in OB animals with respect to healthy normal weight ones (Fig 6A and 6C). It was observed that the locomotor activity in the iNW rabbits decreased by 50% with respect to the iNW group at 63 days post-infestation. The iOB group also decreased by 48% from 7 days post-infestation with respect to the NW group, and by 71.3% at 63 days (Fig 6B). In the exploratory activity, the iOB group had a 72.4% decrease at 21 days post-infestation that was maintained until 63 days with 74% less than the NW group, while the iNW and iOB groups behaved similarly to the NW (Fig 6D). Chin rub marking behavior did not show changes in the OB group, while in both infested groups (iNW and iOB) it decreased (Fig 6E). It began to decrease by 55% in the iOB group at day 14, and decreased to 92% at day 63 compared to the NW group; in the iNW group it decreased by 65% at 21 days and was sustained until day 63 post-infestation. No differences were observed between the NW and OB groups (Fig 6F).

### Productive parameters of does fed a balanced or obesogenic diet, non-infected or infected with *P*. *cuniculi*

**Fig 7:**
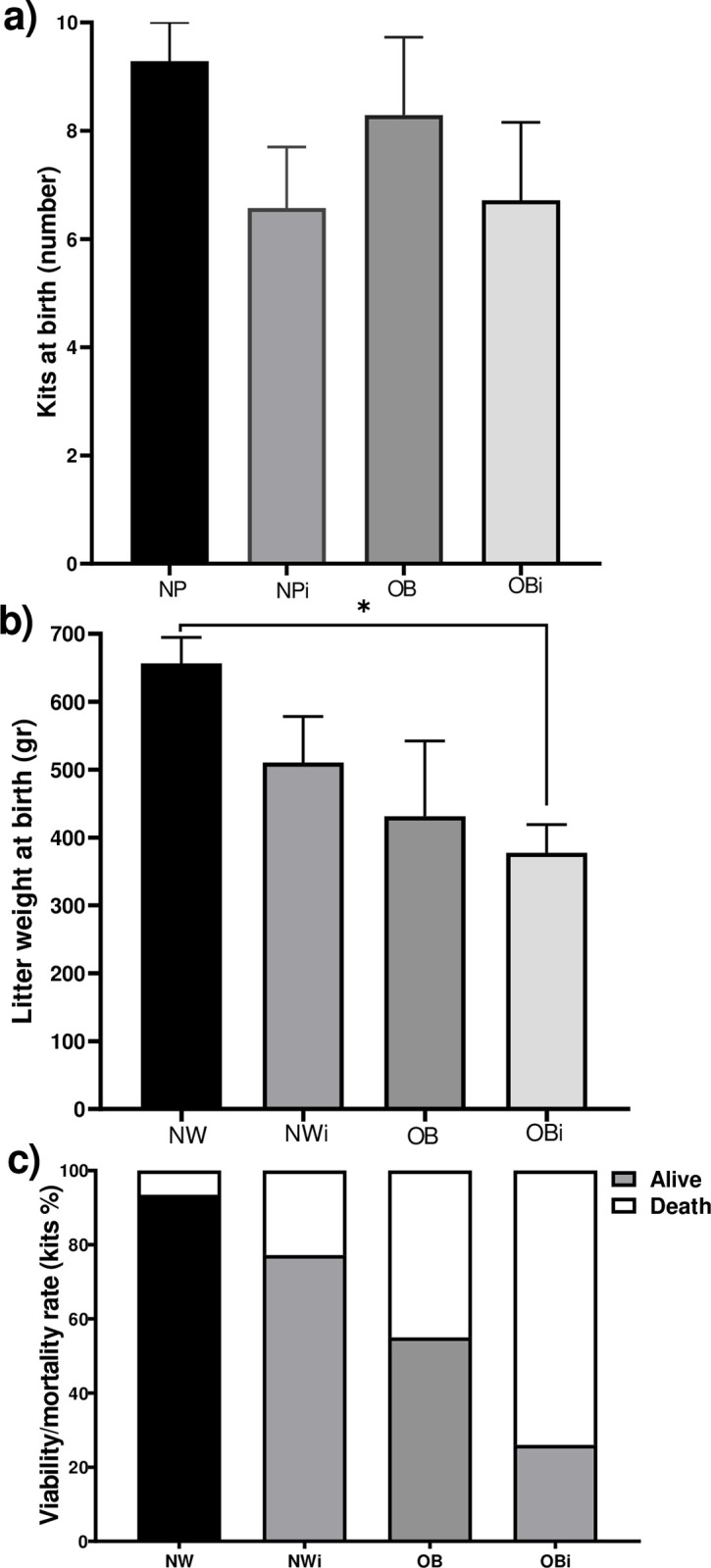
Productive parameters at birth. a) Litter size, b) Litter weight, c) Viability rate of kits. Mean±EE, *P≤0.05, ANOVA, Tukey post-hoc test.

In the productive parameters, the total number of kits at birth did not have a difference between any of the groups, only a trend of 30% less was observed in the iNW and iOB groups compared to the NW group (Fig 7A). In the weight of the litters at birth, the analysis showed a difference between the NW group and the iOB (377.5±41.5 gr), where the iOB had 42.5% less (Fig 7B). Among the live-born kits, a difference of 48.34% was observed between the iOB group compared to the NW group (4.5±1 vs 8.71±0.68, Fig 7C).

**Fig 8:**
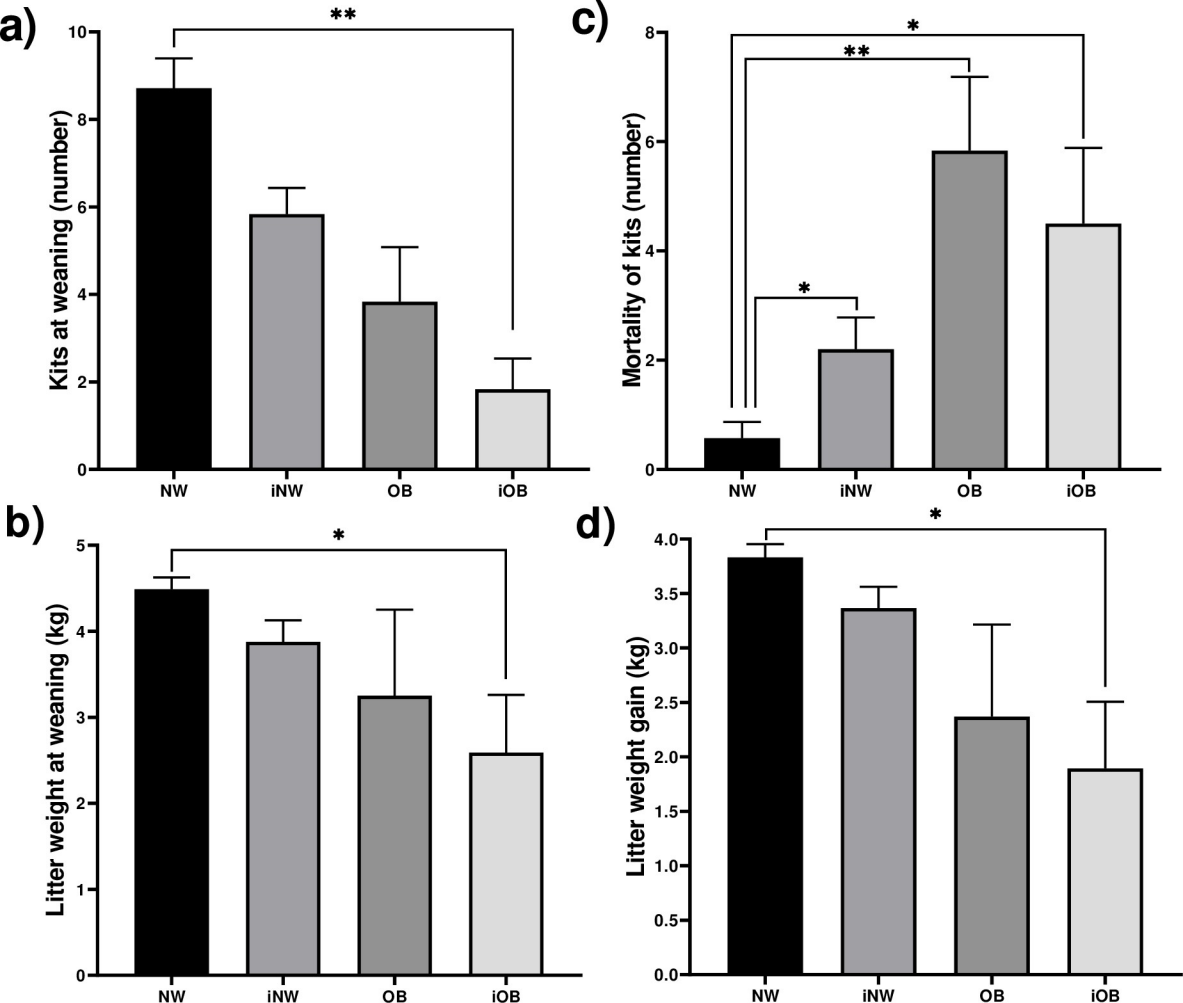
Productive parameters at weaning. a) Litter size, b) Litter weight, c) Mortality of kits, d) Litter weight gain. Mean±EE, *P≤0.05, **P≤0.01. Kruskal-Wallis test and Dunn´s test.

The number of kits at weaning had a decrease of 80% in the iOB group (Fig 8A), while the analysis of litter weights at weaning (Fig 8B) revealed a difference of 1.9 kg between the control and the iOB groups, which represents 42.2% less weight in the litters of the iOB compared to the NW group (2.59±0.67 vs 4.48±0.13). In the number of dead kits in the period from birth to weaning (Fig 8C), there was a higher number in the OB group, representing 90.23% (5.83±1.35), and in the iOB 87.34% (4.50±1.38) with respect to the control group (0.57±0.29). The weight gain in the period from birth to weaning was 50% less in the iOB animals compared to the NW group.

### Qualitative and quantitative assessment of the degree of infection with *P*. *cuniculi*

**Fig 9:**
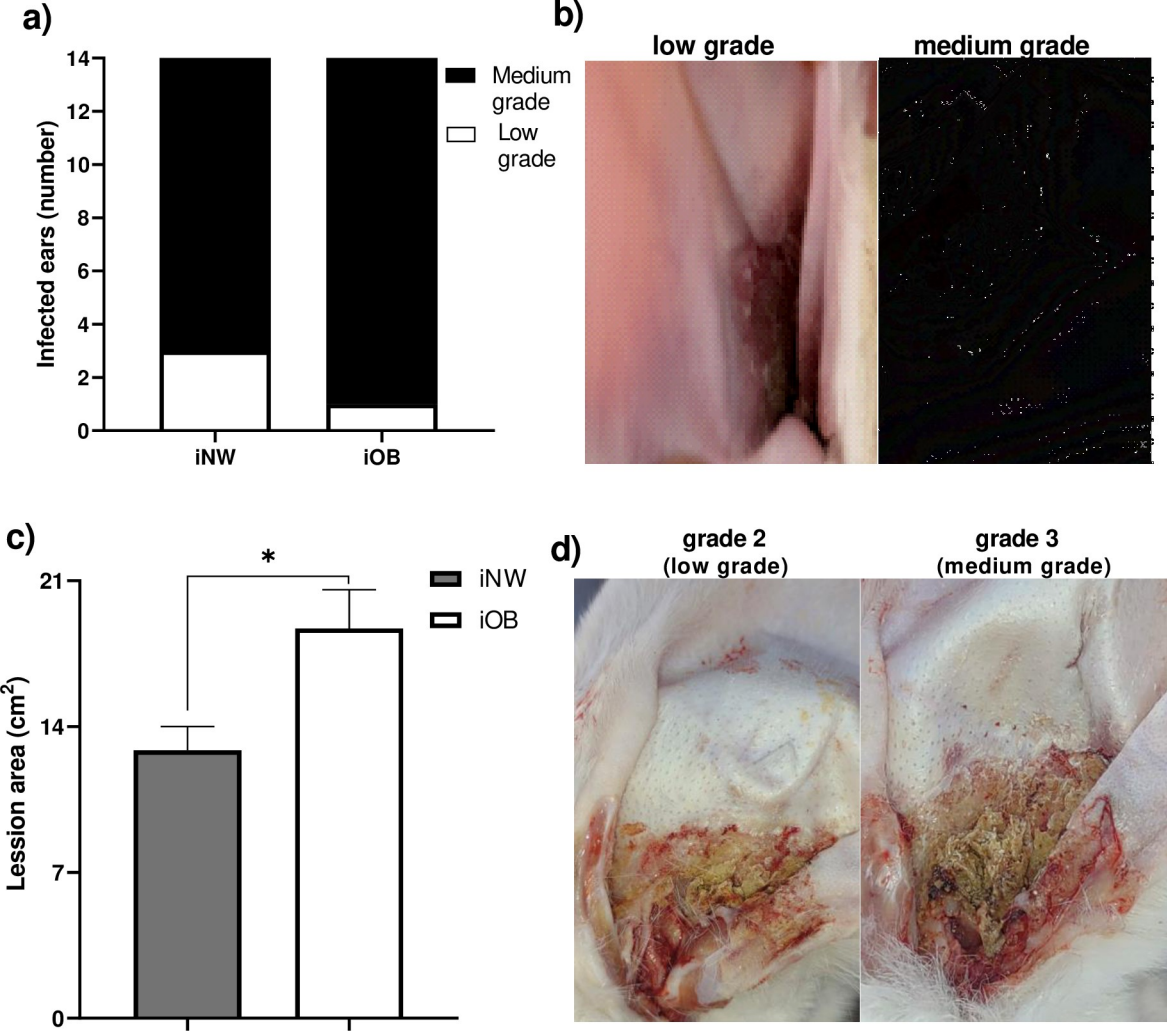
Qualitative and quantitative evaluation of infection with *P*. *cuniculi* in rabbits. a) Number of infested ears and rate of different infestation degree in all the ears, evaluated qualitatively (black bars = medium degree, white bars = low degree), b) Representative image of the degree of infection *in vivo*, c) Degree of infestation (area of the lesion) measured quantitatively, d) Representative image of the lesion at necropsy. Mean±EE, *P≤0.05. Paired student’s T test.

In the evaluation of the lesion caused by the infestation with *P*. *cuniculi*, all the does, both in the NW group and in the OB group, developed infection in both ears, which could be visually appreciated through the evaluation with the otoscope (Fig 9A and 9B), and it was quantitatively confirmed at sacrifice using a squared plastic film (Fig 9C and 9D). In the qualitative evaluation, in the infested NW group, 3 of the ears (22%) had a low degree of infection and 11 had a medium degree of infection (78%), while in the OB, 1 ear presented low grade (8%) and 13 medium grade (92%) (Fig 9A). In the quantitative analysis of the lesion area caused by the *P*. *cuniculi* mite, 31.6% more infection was observed in the iOB group (18.71±1.8 cm2) in relation to the NW group (12.8±1.1 cm2), (Fig 9C and 9D).

## Discussion

Our goal was to analyze the influence of obesity on the infection degree with *P*. *cuniculi* and on behavior and production. Both, infection and obesity caused alterations on productive and behavioral parameters, and the disturbances were magnified by the comorbidity. Also, we found higher susceptibility to infection in the obese does.

### Obesity in rabbits

The criteria for defining obesity in rabbits, as in most animals, are commonly based on qualitative criteria, considering the body condition through the external morphology of the animal and palpation of bones and body fat [20], which can be subjective and requires training. It has also been proposed to quantify body weight to define obesity criteria where it is proposed that rabbits with a weight greater than 10% of the expected weight are overweight and those with a weight greater than 15% are obese [20]. More precisely, it has been proposed to assess overweight and obesity through the use of indices that allow the quantitative estimation of these pathologies. Sweet et al. [10] proposed the inclusion of quantitative two-dimensional measurements estimating adipose tissue mass in rabbits, measuring distal forelimb length (DFL) or vertebral length (VF). They preliminary validated and established limit values for underweight, ideal condition and obese rabbits, but not for overweight animals. In our study we obtained the relationship between weight and DFL (BMI) or weight and BV (ZI), analyzing the two indices proposed by Sweet et al. to estimate obesity in rabbits fed an obesogenic diet.

In our obesity model, we considered a 15% greater body weight in relation to the weight of the control rabbits (NW) to establish that the rabbits were obese. At 42 days after administration of the obesogenic diet we observed an average increase of 15% in weight with 4±0.07 kg (from 3.750 to 4.570 kg) in the OB rabbits, with a BMI of 0.266±0.004 and an IZ of 0.097±0.001, coinciding with the values proposed by Sweet et al. which were >3.5 kg, BMI 0.22–0.47 and IZ 0.072–0.16 to classify them as obese rabbits. In this way we collaborate in the validation of the zoometric indices BMI and ZI proposed for obese animals. At 42 days, the NW animals in this study had a weight of 3.39±0.04 kg (from 3.100 to 3.640 kg), a BMI of 0.219±0.008 and a ZI of 0.081±0.0009, both indices coinciding with the ranges proposed by Sweet et al. [10], which had BMI from 0.16 to 0.23 and ZI from 0.050 to 0.086, but it did not coincide with the weight proposed by them, which was from 2.5 to 3.5 kg. This difference is due to the fact that the weight in rabbits is dependent on the breed, size, sex and age, thus, while in Sweet’s study they have heterogeneous groups of rabbits, in this study we have a homogeneous group, which allows us to establish the specific values of young adult female rabbits of the New Zealand breed. It should be noted that the ranges in Sweet’s study overlap between the values proposed for NW and OB animals, while the ranges in our study are defined for each classification.

### Infection in obese animals affects behavior

Regarding the effect of the ectoparasite-obesity-host triad on productive and behavioral parameters, obesogenic diets in which fat intake is increased have been evaluated in other species such as the mouse, where it has been observed that the induction of obesity was dependent on the mouse strain, since BALB/c mice were resistant to the induction of obesity, while C57BL/6 mice had rapid weight gain. Obesity induced a decrease in the activity of obese mice without finding alterations in other ethological parameters measured in open field, elevated plus maze, social interaction and hotplate [21]. The results observed in mice suggest that obesity-dependent alterations are strain-dependent, and according to the multiple alterations that we observed in rabbits, behavior is also dependent on the species under study [22].

In the current study, locomotor activity decreased in the group of obese rabbits, which coincides with what was observed in species such as the mouse; It is important to highlight that the greatest effect translated into the decrease of the activity corresponded to the animals with the comorbidity ectoparasitism and obesity; in this sense, it has previously been documented that ectoparasites in domestic animals have a direct effect, that is, other pathogens that enter the host using the ectoparasite as a vector are excluded, attributing the changes to the presence of the parasite itself, observed through economic loses like the loss of weight, decrease in milk, eggs, meat, skin or wool production, fetal abortions or death [23].

Here, a decrease in voluntary consumption was observed after 35 days of infestation in the iNW and iOB groups, specifically the decrease was more pronounced in the iOB by 55%, compared to the OB; this decrease has also been observed in cattle when infected by ticks. It has been observed that ticks are capable of releasing toxins, which are capable of inducing anorexia [23]. It is likely that the effect observed in the decrease in voluntary consumption could be due to some toxin secreted by *P*. *cuniculi*, aspects that could be verified experimentally in the future. On the other hand, it has been reported that infestation with *P*. *cuniculi* induces the expression of cytokines such as interleukin 6, 8 and transforming growth factor-β1 in addition to prostaglandin E2 in peripheral blood [6]. Specifically, the increase in serum concentrations of IL 6, together with other chemical mediators, has been associated with anorexia nervosa, which is characterized by weight loss combined with alterations in the immune and neuroendocrine system [24, 25]; this pathology has a certain relationship with what was observed in the iNW and iOB experimental groups. It is also important to consider that the immune system is an organic system requiring large amounts of energy, thus, the host must invest energy in defending itself against infestation by *P*. *cuniculi*, which in addition to altering the behavior, impacts their voluntary consumption, body weight, body mass and zoometric indices.

A decrease in chin rubbing behavior was observed in the infected normal weight group of does. Our observations coincide with what was previously reported in males, since the effect of parasitic infections on chin rubbing behavior has been observed with the ectoparasite *P*. *cuniculi*, where the frequency of this behavior is reduced in rabbits infested both acutely and chronically, specifying that the decrease in this behavior begins from the four days post infestation [15]. It has also been described that *T*. *pisiformis* metacestode, which is an internal parasite, reduced chinning behavior by 25% after 16 days of infection, while with *P*. *cuniculi* in obese animals, the reduction was greater than 55% at 14 days post infestation [4]. This decrease could be attributed to the increase in cortisol, which has been reported increased in rabbits infected with *P*. *cuniculi*, since cortisol can cause a decrease in the expression of estrogens that would be reflected in the diminished activity of chin marking [4]; however, serum cortisol levels are not known in the obesity-infection comorbidity with *P*. *cuniculi*.

The findings of the current study and those previously referred reflect the importance of the study of ethoparasitology, understood as the behavioral modifications that are observed when parasites infect their hosts. These modifications can be measured quantitatively and objectively, and will be modified depending on various factors, including comorbidities such as obesity.

### Obesity affects the susceptibility to infection in animals

Here, it was observed that the infestation evaluated qualitatively did not show statistical differences between the normal weight and obese groups, but a tendency to a higher degree is observed in the iOB (92.86%) compared to the iNW (78.6%), suggesting that obesity generate a susceptibility to the degree of injury caused by acarosis. In the quantitative measurement of the extent of the injury, the obese rabbits did have a noticeable higher injury (iOB = 18.71±1.86) compared to the (iNW = 12.8±1.1), suggesting strongly that obesity causes susceptibility to increased injury by *P*. *cuniculi*. We conclude that quantitative postmortem evaluation is more accurate and reliable than qualitative evaluation and should be recommended when possible. In studies with male rabbits, a greater number of *T*. *pisiformis* metacestodes was found in the infected obese group compared to the normal weight group, while in the normal weight group the number of hepatic granulomas was greater, which are considered a defense mechanism against the development of metacestodes, suggesting that the immune response against the pathogen is affected by obesity [26]. On the other hand, studies performed in obese mice infected with *S*. *mansoni* cercariae and *T*. *spiralis*, suggest that chronic infection decreases and that molecules derived from parasitosis protect against metabolic disorders caused by obesity, by inducing a Th2 immune response [27, 28]. These results indicate that obesity may have a different impact in a parasite and host-dependent manner.

### Parasites and obesity affect reproduction in males and females

*Psoroptes cuniculi* causes a negative impact on two important productive parameters that are offspring at weaning and daily weight gains. With respect to the effects of parasites on reproduction in animals, a phenomenon known as parasitic castration has been characterized, a term rarely used in medicine perhaps because it can be understood ambiguously and equated to the removal of the gonads. However, in parasitology we understand parasitic castration as the partial destruction or alteration of gonadal or reproductive tissue, alteration of sexual behavior, hormonal balance or other modification that results in a partial or total reduction in the reproduction of the host [29]. Thus, *T*. *pisiformis* metacestodes has the capacity to reduce litter size by 40% and embryo implantation, and also induces an increase in progesterone in infected rabbits [4, 30]. In this work, obesity reduces litters by 45%, in a similar way to infection by *T*. *pisiformis*, while infection with *P*. *cuniculi* affects 23%, and the impact of comorbidity seems to have an additive effect, since it reduces litters by 74%, putting the financial viability of rabbit farms at risk.

It is important to define the mechanisms by which these effects are induced, for which further studies are needed, like determining the concentrations of hormones progesterone and estradiol, or whether there is tissue damage in structures such as the ovary and uterus, in addition to accomplish embryo implantation studies focused on elucidate the possible mechanisms involved in the reduction of the reproductive capacity of rabbits.

## Supporting information

S1 Data Raw data obtained through the experiment, from which the mean±SE were obtained to construct Figs 1–9. (XLSX)

S1 Table Mean data contained in Figs 1–9 showing the differences between groups and the differences over time. (DOCX)
