# Supplementary material for: Effect of infestation with Psoroptes cuniculi on reproduction and behavior of obese rabbit does (Oryctolagus cuniculi)
Source: PLoS One. 2024 Aug 28;19(8):e0307803. doi: 10.1371/journal.pone.0307803 (PMC11356430; doi:10.1371/journal.pone.0307803)
Supplement: S1 Table — (DOCX) [file pone.0307803.s002.docx]

**Body weight (Fig 4b)**

Table 4b. Body weight for 63 days post-infestation with *P. cuniculi* in rabbit does

| **Day** | **NW** | **iNW** | **OB** | **iOB** |
| --- | --- | --- | --- | --- |
| 0 | 3.5±0.06^a1^ | 3.5±0.05^a1^ | \|  \| \| --- \|   4.2±0.12^b1^ | 4.1±0.11^b^ |
| 7 | 3.6±0.06^a^ | 3.5±0.04^a1^ | 4.4±0.13^b1^ | 4.1±0.11^b^ |
| 14 | 3.7±0.06^a^ | 3.4±0.06^a1^ | 4.5±0.13^b1^ | 4.2±0.11^b^ |
| 21 | 3.8±0.08^a^ | 3.5±0.02^b1^ | 4.8±0.11^ab2^ | 4.3±0.12^abc^ |
| 28 | 3.8±0.07^a^ | 3.7±0.05^a1^ | 4.7±0.08^b2^ | 4.3±0.10^ab^ |
| 35 | 3.7±0.03^a^ | 3.1±0.05^b2^ | 4.9±0.06^ab2^ | 4.2±0.02^abc^ |
| 42 | 3.8±0.03^a^ | 3±0.03^b2^ | 5.1±0.08^ab2^ | 4.2±0.02^abc^ |
| 49 | 3.9±0.05^a2^ | 3±0.03^b2^ | 5±0.11^ab2^ | 4.3±0.02^abc^ |
| 56 | 3.9±0.05^a2^ | 2.9±0.06^b2^ | 5.2±0.06^ab12^ | 4.3±0.04^abc^ |
| 63 | 3.9±0.08^a2^ | 2.8±0.07^b2^ | 5.3±0.05^ab12^ | 4.5±0.08^abc^ |

NW= normal weight, iNW= infected normal weight, OB= obese, iOB= infected obese

^a,b,c^ Different letters indicate differences between columns.

^1,2^ Different numbers indicate differences between rows.

P≤0.05

**Voluntary intake (Fig 4d)**

Table 4 d. Voluntary intake for 35 days post-infestation with *P. cuniculi* in rabbit does

| **Day** | **NW** | **iNW** | **OB** | **iOB** |
| --- | --- | --- | --- | --- |
| 0 | 170±6.5^a1^ | 130±6.5^b1^ | 104.7±4.8^ab1^ | 105±4.3^ab^ |
| 7 | 167.7±8.3 ^a1^ | 128.5±6.6 ^b1^ | 107.5±6.8 ^b1^ | 104.4±3.8 ^b^ |
| 14 | 193.4±9.6 ^a1^ | 137.2±3.6 ^b1^ | 107.2±4.2 ^ab1^ | 120.8±4.4 ^ab^ |
| 21 | 239.8±9 ^a2^ | 187±13.7 ^b2^ | 157.1±14 ^b2^ | 117.5±6.9 ^ab^ |
| 28 | 259±13 ^a2^ | 201.5±9.6 ^b2^ | 153.1±7.4 ^ab2^ | 111.8±7.5 ^ab^ |
| 35 | 273±5 ^a2^ | 198.4±5.1 ^b2^ | 158±7.9 ^ab2^ | 101.7±3.3 ^abc^ |

NW= normal weight, iNW= infected normal weight, OB= obese, iOB= infected obese

^a,b,c^ Different letters indicate differences between columns

^1,2,3^ Different numbers indicate differences between rows.

P≤0.05

**Body mass index (Fig 5b)**

Table 5b. Body Mass Index during 63 days post-infestation with *P. cuniculi* in rabbit does

| **Day** | **NW** | **iNW** | **OB** | **iOB** |
| --- | --- | --- | --- | --- |
| 0 | 0.20±0.004^a^ | 0.20±0.003^b1^ | 0.27±0.003^ab1^ | 0.26±0.007^ab^ |
| 14 | 0.20±0.004^a^ | 0.19±0.004^b1^ | 0.29±0.008^ab^ | 0.26±0.007^ab^ |
| 28 | 0.21±0.003^a^ | 0.19±0.002^b1^ | 0.30±0.009^ab^ | 0.28±0.011^ab^ |
| 42 | 0.21±0.005^a^ | 0.19±0.005^b^ | 0.30±0.008^ab^ | 0.27±0.008^ab^ |
| 56 | 0.20±0.003^a^ | 0.19±0.004^b^ | 0.30±0.007^ab^ | 0.27±0.005^abc^ |
| 63 | 0.21±0.003^a^ | 0.17±0.003^b2^ | 0.32±0.009^ab2^ | 0.26±0.004 |

NW= normal weight, iNW= infected normal weight, OB= obese, iOB= infected obese

^a,b,c^ Different letters indicate differences between columns.

^1,2^ Different numbers indicate differences between rows.

P≤0.05

**Zoometric index (Fig 5d)**

Table 5 d. Zoometric index during 63 days post-infestation with *P. cuniculi* in rabbit does

| **Day** | **NW** | **iNW** | **OB** | **iOB** |
| --- | --- | --- | --- | --- |
| 0 | 0.08±0.001^a1^ | 0.08±0.002^a^ | 0.09±0.002^b1^ | 0.09±0.001^b^ |
| 14 | 0.08±0.001^a^ | 0.07±0.002^b^ | 0.10±0.003^ab1^ | 0.10±0.003^ab^ |
| 28 | 0.08±0.002^a^ | 0.07±0.002^b^ | 0.11±0.003^ab2^ | 0.10±0.003^abc^ |
| 42 | 0.09±0.002^a^ | 0.07±0.001^b^ | 0.11±0.003^ab2^ | 0.10±0.003^abc^ |
| 56 | 0.08±0.001^a^ | 0.07±0.002^b^ | 0.11±0.001^ab2^ | 0.10±0.001^abc^ |
| 63 | 0.09±0.001^a2^ | 0.07±0.003^b^ | 0.12±0.003^ab2^ | 0.10±0.001^abc^ |

NW= normal weight, iNW= infected normal weight, OB= obese, iOB= infected obese

^a,b,c^ Different letters indicate differences between columns.

^1,2^ Different numbers indicate differences between rows.

P≤0.05

**Locomotor activity (Fig 6b)**

Table 6b. Locomotor activity during 63 days post-infestation with *P. cuniculi* in rabbit does

| **Day** | **NW** | **iNW** | **OB** | **iOB** |
| --- | --- | --- | --- | --- |
| 7 | 51±6^a^ | 31±2 | 37±3^1^ | 26±4^b^ |
| 14 | 53±5^a^ | 34±2 | 40±4^a1^ | 24±4^b^ |
| 21 | 45±4^a^ | 31±3^a^ | 33±3^a^ | 17±3^b^ |
| 49 | 39±5^a^ | 24±2^a^ | 23±5 | 12±2^b^ |
| 56 | 36±5^a^ | 22±4 | 19±6^2^ | 12±3^b^ |
| 63 | 43±6^a^ | 21±3^b^ | 23±7 | 12±3^b^ |

NW= normal weight, iNW= infected normal weight, OB= obese, iOB= infected obese

^a,b^ Different letters indicate differences between columns

^1,2^ Different numbers indicate differences between rows.

P≤0.05

**Exploratory activity (Fig 6d)**

Table 6d. Exploratory activity during 63 days post-infestation with *P. cuniculi* in rabbit does

| **Day** | **NW** | **iNW** | **OB** | **iOB** |
| --- | --- | --- | --- | --- |
| 7 | 5±1 | 5±1 | 3±1 | 2±0.3 |
| 14 | 6±1 | 4±1 | 3±1 | 2±0.5 |
| 21 | 5±1^a^ | 4±1 | 4±1 | 1±0.5^b^ |
| 49 | 4±1^a^ | 5±1 | 2±0.4 | 1±0.5^b^ |
| 56 | 5±1^a^ | 3±1 | 2±1 | 2±0.5^b^ |
| 63 | 5±1^a^ | 4±1 | 3±1 | 1±05^b^ |

NW= normal weight, iNW= infected normal weight, OB= obese, iOB= infected obese

^a,b^ Different letters indicate differences between columns.

P≤0.05

**Chinning (Fig 6f)**

Table 6f. Chinning during 63 days post-infestation with *P. cuniculi* in rabbit does

| **Day** | **NW** | **iNW** | **OB** | **iOB** |
| --- | --- | --- | --- | --- |
| 7 | 16±3 | 10±2 | 15±4 | 10±1 |
| 14 | 17±1^a^ | 10±2 | 13±3 | 7±1^b^ |
| 21 | 18±3^a^ | 6±1^b^ | 14±3 | 6±0.2^b^ |
| 49 | 10±1^a^ | 4±1^b^ | 10±4 | 3±0.5^b^ |
| 56 | 14±3^a^ | 3±1^b^ | 7±4 | 3±1^b^ |
| 63 | 15±2^a^ | 5±2 | 11±4 | 1±0.4^b^ |

NW= normal weight, iNW= infected normal weight, OB= obese, iOB= infected obese

^a,b^ Different letters indicate differences between columns

P≤0.05
